# Evaluation of Parenteral Vitamin C's Effectiveness in Critically Ill Patients: A Systematic Review and Critical Appraisal

**DOI:** 10.7759/cureus.67184

**Published:** 2024-08-19

**Authors:** Pallav V Thakare, Sagar S Gaurkar, Sandip A Mohale, Gopikishan Bharadia, Sourya Acharya

**Affiliations:** 1 Internal Medicine, Jawaharlal Nehru Medical College, Datta Meghe Institute of Higher Education and Research, Wardha, IND; 2 Otolaryngology - Head and Neck Surgery, Jawaharlal Nehru Medical College, Datta Meghe Institute of Higher Education and Research, Wardha, IND; 3 Internal Medicine, Vivekanand Hospital, Latur, IND

**Keywords:** clinical outcomes, systematic review, parenteral administration, severe infections, sepsis, vitamin c

## Abstract

Vitamin C, a key nutrient with potent antioxidant and immunomodulatory properties, has been explored for its therapeutic potential in treating severe infections, particularly sepsis. This systematic review aims to evaluate the effectiveness of parenteral vitamin C in improving clinical outcomes in patients with severe infections. A comprehensive search of several databases, including PubMed, EMBASE, and the Cochrane Library, was conducted for studies published between January 2000 and June 2024. Included were randomized controlled trials, observational studies, and case reports that examined the use of parenteral vitamin C in adult patients with severe infections. Data extracted included study design, sample size, intervention specifics, and clinical outcomes. Quality was assessed using tools appropriate to each study design, such as the Cochrane Risk of Bias Tool and the Newcastle-Ottawa Scale. The review included nine studies with diverse methodologies. While individual studies reported benefits such as improved immune function and reduced oxidative stress, larger systematic reviews and meta-analyses did not demonstrate a significant reduction in mortality. The results indicate that while parenteral vitamin C may improve certain biochemical and physiological parameters, these improvements do not consistently translate into enhanced survival or substantial clinical benefits. Parenteral vitamin C shows potential in modulating immune response and reducing oxidative damage in severe infections. However, its impact on key clinical outcomes like mortality and long-term recovery remains uncertain. This review highlights the need for further high-quality, randomized controlled trials to clarify vitamin C's role in managing severe infections and define optimal therapeutic protocols.

## Introduction and background

Vitamin C, or ascorbic acid, is a crucial micronutrient known for its extensive role in human health. It is particularly an antioxidant and an essential factor in collagen synthesis, wound healing, and immune system function [1]. Vitamin C helps neutralize harmful free radicals as an antioxidant, protecting cells from oxidative stress. It is also vital for the biosynthesis of collagen. This protein is a fundamental component of connective tissues, aiding in wound healing and maintaining the integrity of skin, blood vessels, and bones [2]. Furthermore, vitamin C plays a critical role in the proper functioning of the immune system, enhancing the activity of various immune cells and promoting the body's defense mechanisms against infections [3].

Vitamin C has garnered attention in severe infections, especially sepsis, due to its potential to mitigate oxidative stress and bolster immune defenses [4]. According to the Centers for Disease Control and Prevention (CDC), sepsis affects at least 1.7 million adults in the United States each year [4]. Sepsis, a life-threatening condition characterized by a systemic inflammatory response to infection, leads to increased oxidative stress and immune dysfunction [5]. Vitamin C could play a dual role in this setting: as an antioxidant, reducing damage from reactive oxygen species, and as an immune support, enhancing the function of various immune cells [1]. Clinical studies have explored these properties, with some suggesting improvements in patient outcomes such as reduced ventilator dependency and shortened intensive care unit stays when high doses of vitamin C are administered. These findings highlight the therapeutic potential of vitamin C in managing severe infections and improving patient prognosis [6].

The interest in using vitamin C, particularly administered parenterally, stems from its pharmacokinetic properties, which allow higher plasma concentrations to be achieved compared to oral administration, potentially maximizing its therapeutic effects [7]. Parenteral administration of vitamin C is particularly beneficial in critically ill patients, who often have lower plasma levels due to increased metabolic demand and reduced oral intake. By administering vitamin C intravenously, healthcare providers can rapidly elevate plasma levels, which may be crucial during the acute phases of severe infections when timely intervention is essential [8]. This approach not only ensures that sufficient levels of the vitamin are available to exert its antioxidant and immune-boosting effects but also addresses the practical challenges of repletion in patients who may be unable to ingest or absorb sufficient quantities orally [9].

## Review

Study design and protocol

This systematic review adheres to the Preferred Reporting Items for Systematic Reviews and Meta-Analyses (PRISMA) [10] guidelines to ensure clarity, transparency, and rigor in reporting. The aim is to evaluate the effectiveness and safety of parenteral vitamin C administration in severe infections, focusing on outcomes such as survival rates, symptom improvement, and biomarkers indicative of infection and recovery. This methodological approach allows for a structured and comprehensive synthesis of the existing literature, providing a clear overview of the therapeutic potential of vitamin C in clinical settings [10]. The Population, Intervention, Comparison, and Outcomes (PICO) framework for evaluating the effectiveness of parenteral vitamin C in critically ill patients is shown in Table 1.

**Table 1:** The PICO framework for evaluating the effectiveness of parenteral vitamin C in critically ill patients PICO: Population, Intervention, Comparison and Outcomes

Component	Description
Population	Critically ill adult patients with severe infections, including sepsis.
Intervention	Parenteral administration of vitamin C (intravenous or intra-arterial).
Comparison	Standard care without parenteral vitamin C or with other antioxidant therapies; oral vitamin C supplementation (if applicable).
Outcome	Key clinical outcomes include mortality rates, length of hospital stay, symptom improvement, physiological markers of infection (e.g., oxidative stress reduction, immune function enhancement), and adverse events.

Selection criteria

Studies included in this review examined the administration of vitamin C through parenteral routes, specifically intravenous or intra-arterial, in adult patients with severe infections. We included various study designs, such as randomized controlled trials (RCTs), observational studies, case reports, and previous systematic reviews. Crucially, the studies needed to report on measurable clinical outcomes, such as mortality rates, length of hospital stay, symptom management, physiological markers of infection, or adverse effects. The exclusion criteria were clearly defined to isolate the impact of parenteral vitamin C from other forms of supplementation. Studies focusing solely on oral vitamin C supplementation or those combining different supplementation methods without isolating the effects of parenteral administration were excluded. Additionally, non-peer-reviewed articles, conference abstracts, editorials, and expert opinions were omitted from this review. Studies that lacked complete data or failed to report specific outcomes of interest were also excluded. This methodological framework ensures the review is based on peer-reviewed, scientifically valid studies, providing a reliable and robust analysis of parenteral vitamin C’s role in treating severe infections. Through this rigorous selection process, the review aims to offer valuable insights for clinical practice and identify areas needing further investigation.

Search strategy

**Figure 1:**
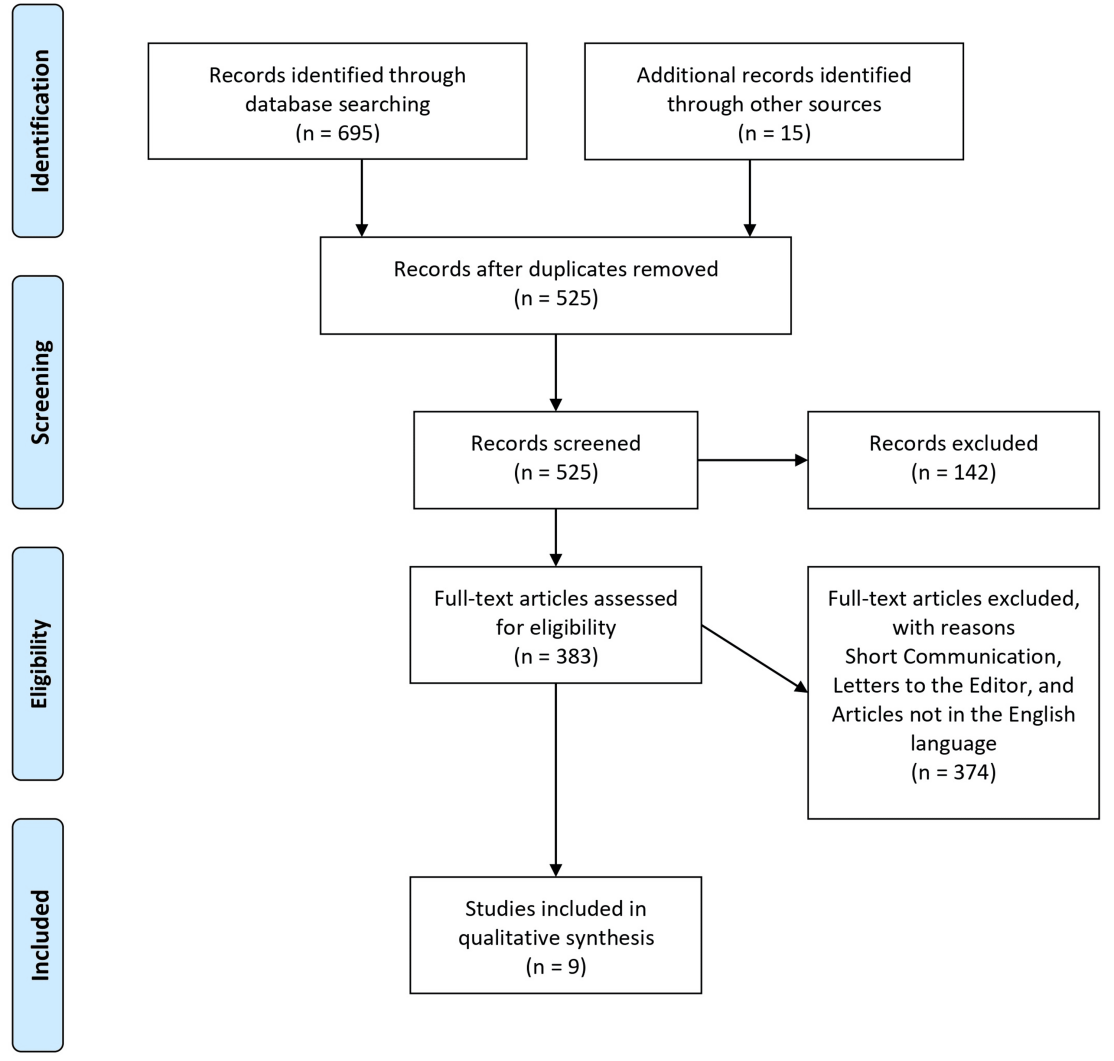
The selection process of articles used in this study Adopted from the Preferred Reporting Items for Systematic Reviews and Meta-Analyses (PRISMA).

The literature search strategy was meticulously planned to include several major databases to ensure a comprehensive collection of relevant studies. We searched PubMed, EMBASE, Web of Science, and the Cochrane Library, targeting publications from January 2000 to June 2024. The search utilized a combination of keywords and phrases such as "parenteral vitamin C," "intravenous vitamin C," "severe infection," "sepsis," and "antioxidant therapy." The reference lists of identified and relevant review articles were manually screened for additional sources to ensure completeness. This broad and thorough search strategy was designed to capture all pertinent data on the subject, minimizing the risk of bias in the review process. Figure 1 illustrates the selection procedure for the papers included in the present study.

Data synthesis and ethical considerations

Data from the included studies were synthesized to evaluate the efficacy and safety of parenteral vitamin C in severe infections. Due to the heterogeneity of study designs and outcomes, we employed a narrative synthesis approach. This method involved summarizing findings across different studies, highlighting key trends, and noting any discrepancies in results. The synthesis aimed to provide a clear overview of the current evidence, elucidating vitamin C treatment's potential benefits and limitations in clinical practice. In conducting this systematic review, ethical considerations were focused on the adherence to ethical standards in the reporting and using data from the original studies. We ensured that all included studies had appropriate ethical clearances and that our review process respected these guidelines. The ethical review also extended to the interpretation of findings, ensuring that recommendations for clinical practice based on this review consider patient safety and informed consent, especially in contexts involving high-dose interventions. This careful consideration helps maintain the review's integrity and supports its applicability in real-world medical settings.

Data extraction and quality assessment

Data extraction for this systematic review was carried out meticulously using a standardized data collection form to ensure uniformity across studies and minimize extraction errors. For each included study, we extracted essential information, including the authors, year of publication, study design, sample size, specifics of the intervention (type, dosage, frequency, and duration of parenteral vitamin C administration), duration of the study, and key outcomes such as mortality, symptom relief, biomarker changes, and adverse events. This detailed data collection allows for a comprehensive comparison and synthesis across studies, facilitating a thorough evaluation of the effects of parenteral vitamin C on severe infections.

The quality of each study included in this systematic review was rigorously assessed using different tools tailored to the specific study designs. RCTs were evaluated with the Cochrane Risk of Bias Tool [11], focusing on randomization, blinding, completeness of outcome data, selective reporting, and other potential biases. Observational studies were appraised using the Newcastle-Ottawa Scale (NOS) [12], which examines the selection of study groups, their comparability, and the ascertainment of exposure or outcome to address potential confounders and biases. Case reports were assessed with the CARE guidelines [13], ensuring comprehensive and clear reporting of clinical information, including medical history, clinical findings, diagnostics, interventions, and outcomes. Systematic reviews were evaluated for methodological quality using A Measurement Tool to Assess Systematic Reviews, version 2 (AMSTAR 2) [14], considering the comprehensiveness of literature searches, justification of excluded studies, the validity of methods used to combine findings, and risk of bias within included studies. These assessment tools collectively ensure that the conclusions drawn from this systematic review are based on robust and reliable data. By meticulously evaluating the quality of each study, we aim to enhance the credibility of our findings and provide a solid foundation for future research and clinical practice. This rigorous approach to quality assessment helps mitigate potential biases and ensures that the insights derived from the reviewed studies are valid and applicable. The quality assessment is shown in Table 2.

**Table 2:** Quality assessment table N/A: Not Applicable; RCT: Randomized Controlled Trial; CARE Guidelines: Case Report Guidelines; AMSTAR 2: A Measurement Tool to Assess Systematic Reviews, version 2

Study Reference	Study Design	Assessment Tool	Criteria Evaluated	Overall Quality Rating
Wilson et al. [15]	Review	N/A	N/A	N/A
Ferrón-Celma et al. [16]	RCT	Cochrane Risk of Bias Tool	Random sequence generation, blinding, outcome data	High
Carr et al. [17]	Case Report	CARE Guidelines	Completeness of case data, clinical detail	Moderate
Lehr et al. [18]	Consensus Meeting	Not applicable	N/A	N/A
Carr et al. [19]	Observational Study	Newcastle-Ottawa Scale	Selection, comparability, outcome ascertainment	High
McGregor et al. [20]	Review	Not applicable	N/A	N/A
Brown et al. [21]	Systematic Review	AMSTAR 2	Multiple domains, including conflict of interest	High
Yanase et al. [22]	Review	Not applicable	N/A	N/A
JamaliMoghadamSiahkali et al. [23]	RCT	Cochrane Risk of Bias Tool	Allocation concealment, blinding, selective reporting	Moderate

Results

The results of this systematic review provide a comprehensive overview of parenteral vitamin C's effectiveness in treating severe infections. A total of nine studies were included in the final analysis, encompassing a diverse range of methodologies and patient demographics. Here, we discuss the main findings and synthesize the outcomes reported across the included studies, and Table 3 displays the characteristics of the articles used in this review.

**Table 3:** Characteristics of included studies N/A: Not Applicable; RCT: Randomized Controlled Trial; SpO_2_: Peripheral Oxygen Saturation

Authors	Type of Study	Sample Size	Intervention	Duration	Results
Wilson et al. [11]	Review	N/A	High-dose parenteral vitamin C	N/A	Potential adjuvant therapy benefits in sepsis
Ferrón-Celma et al. [12]	RCT	20	450 mg/d of parenteral vitamin C	6 days	Anti-apoptotic effects on neutrophils
Carr et al. [13]	Case Report	1 (case study)	Parenteral vitamin C (50 g/session)	N/A	Improved fatigue, pain, and insomnia symptoms
Lehr et al. [14]	Consensus meeting	N/A	High-dose parenteral vitamin C	N/A	Restoration of endothelial function
Carr et al. [15]	Observational study	44	Vitamin C supplementation via enteral and/or parenteral nutrition	4 days	Low vitamin C levels despite standard nutrition, higher in septic shock patients
McGregor et al. [16]	Review	N/A	High-dose parenteral vitamin C	N/A	Reduced oxidative stress and edema in critically ill patients
Brown et al. [17]	Systematic review and meta-analysis	4078	Parenteral vitamin C vs. standard care	N/A	No significant mortality reduction; further studies are needed
Yanase et al. [18]	Review	855	High-dose parenteral vitamin C (75 mg/kg/d)	N/A	No clinical efficacy or harm; further investigation justified
JamaliMoghadamSiahkali et al. [19]	RCT	60	High-dose intravenous vitamin C (6 g daily)	Duration of hospitalization	Improved body temperature and SpO_2_, no significant difference in overall outcomes

Clinical outcomes

Improvement in Clinical Symptoms and Biomarkers

Several studies reported improvements in clinical symptoms and biomarkers using parenteral vitamin C. For instance, Ferrón-Celma et al. [12] A small RCT was conducted with 20 participants, where the administration of 450 mg/day of parenteral vitamin C over six days exhibited anti-apoptotic effects on neutrophils, suggesting an enhancement in immune cell function. Similarly, JamaliMoghadamSiahkali et al. [19] performed an RCT involving 60 participants, finding that high-dose intravenous vitamin C (6 g daily) improved body temperature and peripheral oxygen saturation (SpO_^2^_) levels during the hospital stay. However, it did not significantly affect overall outcomes. Additionally, Carr et al. [15] documented a case report where a single patient experienced improved symptoms of fatigue, pain, and insomnia following sessions of 50 g per session of parenteral vitamin C.

Mortality and Severe Outcomes

The evidence regarding mortality and severe outcomes was less conclusive. Brown et al. [17] conducted a large systematic review and meta-analysis involving 4,078 patients and compared parenteral vitamin C versus standard care. The study found no significant reduction in mortality, indicating that while vitamin C may improve some clinical symptoms, it does not necessarily translate into a survival benefit. Yanase et al. [18] reviewed data from 855 patients treated with high doses of parenteral vitamin C (≥75 mg/kg/day) and reported no clinical efficacy or harm, suggesting that more research is needed to evaluate its impact on survival and other critical outcomes.

Biological Mechanisms and Physiological Effects

Two reviews by Wilson et al. [11] and McGregor et al. [16] highlighted the potential of high-dose parenteral vitamin C as an adjuvant therapy in sepsis. They suggested that vitamin C contributes to the reduction of oxidative stress and may help restore endothelial function, which is crucial in managing severe infections. These biological mechanisms underpin the observed clinical improvements and provide a rationale for further investigation into the therapeutic role of vitamin C in severe infections. The results also showed considerable variability in outcomes, which can be attributed to differences in study design, population demographics, vitamin C dosing regimens, and the clinical settings of the studies. The quality assessment revealed that while some studies, particularly RCTs and systematic reviews, were of high quality, case reports and smaller observational studies had limitations that could affect the generalizability of the findings. The synthesis of the results from the included studies suggests a complex picture. While parenteral vitamin C appears to have some beneficial effects on immune function and symptom management in severe infections, these benefits do not consistently translate into reduced mortality or major changes in clinical outcomes such as the length of hospital stay or recovery rates. High-quality, large-scale RCTs are needed to further elucidate the role of parenteral vitamin C in severe infections, particularly in sepsis, to determine optimal dosing and administration protocols and to confirm its efficacy and safety in these critically ill patient populations.

Discussion

The results of this systematic review highlight the complex role of parenteral vitamin C in managing severe infections. While several studies suggest beneficial effects on specific clinical symptoms and biomarkers, the overall impact on mortality and major clinical outcomes remains uncertain. This inconsistency can be attributed to several factors, including variability in study design, differences in patient populations, and the varying dosages of vitamin C administered.

Immunomodulatory and Antioxidant Effects

Several studies within this review noted improvements in immune function and antioxidant protection due to parenteral vitamin C. Ferrón-Celma et al. (2020) reported that vitamin C exhibits anti-apoptotic effects on neutrophils, suggesting that it may enhance immune cell resilience in the context of infections [12]. This finding supports the known physiological role of vitamin C as a potent antioxidant and a crucial cofactor in collagen synthesis and immune function [24]. Similarly, Wilson et al. (2021) discussed the potential benefits of vitamin C as an adjuvant therapy in sepsis, particularly its role in mitigating oxidative stress [11].

Impact on Clinical Outcomes

Despite these positive biochemical and cellular responses, translating these effects into improved survival rates or significant clinical benefits has proven challenging. For example, Brown et al. (2023) conducted a systematic review and meta-analysis involving 4078 patients, which found no significant reduction in mortality with parenteral vitamin C compared to standard care [17]. This lack of significant mortality benefit is corroborated by Yanase et al. (2022), who also found no clinical efficacy in their review of 855 patients [18]. These findings suggest that while vitamin C may improve physiological and biochemical markers, these improvements do not necessarily correlate with critical patient-centric outcomes like mortality and long-term recovery. The variability in study quality also plays a crucial role in interpreting these results. Higher-quality studies, such as RCTs, provided more reliable data, whereas observational studies and case reports often introduced bias and limitations in generalizability. This highlights the need for well-designed, large-scale RCTs to provide more definitive evidence on the efficacy of parenteral vitamin C in critically ill patients [25,26].

Limitations and future directions

Future research should focus on defining the optimal dosing, timing, and administration routes for vitamin C in different severe infection scenarios. Moreover, understanding patient-specific factors that may influence the response to vitamin C, such as baseline nutritional status and the presence of comorbidities, could tailor therapy more effectively.

## Conclusions

The findings from this systematic review indicate that while parenteral vitamin C can exert positive effects on certain physiological and immunological parameters in severe infections, its impact on critical clinical outcomes such as mortality remains inconclusive. The evidence gathered from various study designs suggests potential benefits in improving symptoms and reducing oxidative stress; however, these improvements do not consistently translate into enhanced survival rates or significant clinical recovery. Given the variability in study results and the limitations associated with different research methodologies, it is evident that further high-quality RCTs are needed to better define the therapeutic role of vitamin C in severe infections. Such research should establish optimized dosing, timing, and administration strategies while considering patient-specific factors that could influence treatment efficacy. Ultimately, while parenteral vitamin C holds promise as an adjunct therapy in treating severe infections, robust evidence is required to substantiate its clinical benefits and guide its use in medical practice.
